# The sea lamprey has a primordial accessory olfactory system

**DOI:** 10.1186/1471-2148-13-172

**Published:** 2013-08-17

**Authors:** Steven Chang, Yu-Wen Chung-Davidson, Scot V Libants, Kaben G Nanlohy, Matti Kiupel, C Titus Brown, Weiming Li

**Affiliations:** 1 Department of Fisheries and Wildlife, Michigan State University, 480 Wilson Road, East Lansing, MI 48824, USA; 2 Diagnostic Center for Population and Animal Health, Michigan State University, 4125 Beaumont Road, East Lansing, MI 48824, USA; 3 Department of Computer Science and Engineering, Michigan State University, 428 S. Shaw Lane, East Lansing, MI 48824, USA; 4 Department of Microbiology and Molecular Genetics, Michigan State University, 567 Wilson Road, East Lansing, MI 48824, USA

## Abstract

**Background:**

A dual olfactory system, represented by two anatomically distinct but spatially proximate chemosensory epithelia that project to separate areas of the forebrain, is known in several classes of tetrapods. Lungfish are the earliest evolving vertebrates known to have this dual system, comprising a main olfactory and a vomeronasal system (VNO). Lampreys, a group of jawless vertebrates, have a single nasal capsule containing two anatomically distinct epithelia, the main (MOE) and the accessory olfactory epithelia (AOE). We speculated that lamprey AOE projects to specific telencephalic regions as a precursor to the tetrapod vomeronasal system.

**Results:**

To test this hypothesis, we characterized the neural circuits and molecular profiles of the accessory olfactory epithelium in the sea lamprey (*Petromyzon marinus*). Neural tract-tracing revealed direct and reciprocal connections with the dorsomedial telencephalic neuropil (DTN) which in turn projects directly to the dorsal pallium and the rostral hypothalamus. High-throughput sequencing demonstrated that the main and the accessory olfactory epithelia have virtually identical profiles of expressed genes. Real time quantitative PCR confirmed expression of representatives of all 3 chemoreceptor gene families identified in the sea lamprey genome.

**Conclusion:**

Anatomical and molecular evidence shows that the sea lamprey has a primordial accessory olfactory system that may serve a chemosensory function.

## Background

A dual olfactory system is thought to be unique to tetrapods. The two distinct sensory epithelia of this system, the main and the vomeronasal olfactory epithelia, heterogeneously express families of chemoreceptor genes, with some overlap [1]. These epithelia have anatomically distinct projections to different parts of the forebrain. These dichotomous molecular and anatomical profiles led to the hypothesis that the VNO is specialized to detect pheromones [2-6] whereas other research has suggested overlapping functions for the main olfactory system and VNO [7-11]. Amphibians were thought to be the earliest evolving animals with a complete VNO [12,13], however recent work has shown that lungfish have a vomeronasal system [14]. It should be noted that although they do not possess a canonically recognized VNO system, molecular components of a VNO system exist in elephant shark [15] and teleost fish [16,17]. Therefore, the vomeronasal system is presumed to have evolved after the main olfactory system in the vertebrate lineage [18].

Although a distinct vomeronasal system has not been identified in fish [19,20], a recent study has found a vomeronasal system in a sister group to tetrapods, the lungfish [14,21]. Moreover, molecular components of a vomeronasal system have been identified in a basal vertebrate, the sea lamprey (*Petromyzon marinus*) [15,22]. Although fish have only one recognized olfactory epithelium, Dulka (1993) [19] suggested a functional division of the primary olfactory pathway in goldfish that may be analagous to the output neurons from the MOE and VOE in tetrapods. Interestingly, the sea lamprey, like tetrapods, has two separate and distinct olfactory epithelia. The AOE was discovered by in 1887 by Scott [23], but its function had eluded description. In 2009, Ren et al. showed that lamprey AOE is lined with a simple cuboidal ciliated epithelium and projects to the medial olfactory bulb [24]. In addition, another structure with elusive function in the sea lamprey brain, the dorsomedial telencephalic neuropil (DTN) [25], is located in a similar position to the tetrapod AOB. The sea lamprey DTN is dorsomedially situated, immediately caudal to the olfactory bulb, receives input from the olfactory bulb and projects to the hypothalamus [26,27].

We hypothesized that the AOE coupled with the DTN comprise a primitive form of the vomeronasal system in the vertebrate lineage. We reasoned that if the AOE was chemosensory, it should express at least some of the chemoreceptor (CR) genes encoded in the lamprey genome [22]. We further reasoned that the AOE projects to a separate telencephalic region, possibly the DTN. Here we present evidence that AOE expresses all known families of lamprey CR genes and projects to the DTN. We conclude that the AOE-DTN-hypothalamic pathway in lamprey is a partial segregation of the olfactory pathway, which suggests that the components of a vomeronasal system may have been in place in this basal vertebrate.

## Results

### AOE projects to the DTN and other telencephalic areas

**Figure 1:**
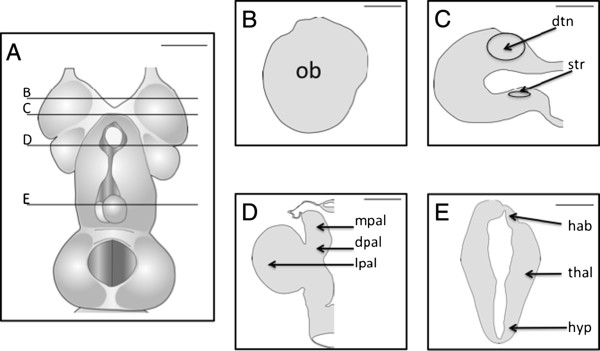
**Diagram of dorsal view of adult lamprey brain and coronal plane slices. A**, The adult lamprey brain is shown in dorsal view with lines representative coronal sections indicated by lines and letters. **B**, Olfactory bulb. **C**, Rostral telencephalon with the dorsomedial telencephalic neuropil and striatum. **D**, Telencephalon with lateral, medial and dorsal pallia. **E**, Caudal telencephalon with habenula, thalamus, hypothalamus. Scale bar in all pictures is 100 μm. dpal: dorsal pallium; dtn: dorsomedial telencephalic neuropil; hab: habenula; hyp: hypothalamus; lpal.: lateral pallium; mpal.: medial pallium; ob: olfactory bulb; str: striatum; thal: thalamus.

**Figure 2:**
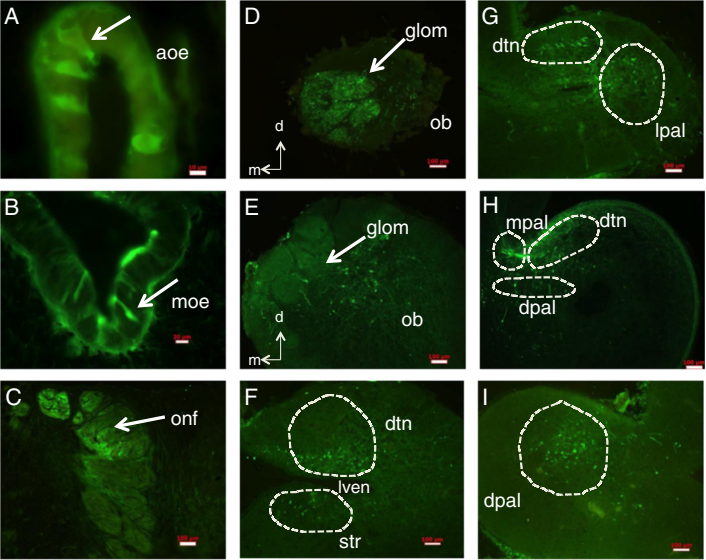
**Anterograde and retrograde connections of the lamprey accessory olfactory organ.** Biocytin injections were made to AOE vesicles and representative pictures are shown here. **A**, following dye loading, short stout cells with cilia extending into the lumen of the AOE are visible (red arrows). Scale bar: 10 μm. **B**, olfactory sensory neurons with long thin axons are retrogradely labeled in the valleys of main olfactory lamellae (red arrows). Scale bar: 20 μm. **C**, the dorsal half of the nerve is preferentially labeled, reflecting the dorsal pathway of axons from the AOE to the telencephalon. Cell bodies and fibers are seen in the medial **(D)** and central **(E)** part of the olfactory bulb. The ventral portion of the DTN and the striatum **(F)** have short thin fibers and cell bodies. The DTN has coarse, thick fibers and the lateral pallium has short, coarse fibers and cell bodies **(G)**. A bundle of thick fibers is visible from the medial pallium to the DTN **(H)** and cell bodies are visible in the dorsal pallium and the ventral border of the DTN. The lateral pallium has a grouping of cell bodies and a mixed population of thin and thick fibers **(I)**. aoe: accessory olfactory epithelium; dpal: dorsal pallium; dtn: dorsomedial telencephalic neuropil; glom: olfactory glomerulus; lpal: lateral pallium; lven: lateral ventricle; ob: olfactory bulb; onf : olfactory nerve fascicle; str: striatum. Scale bars for C-I: 100 μm.

Figure 1 is an atlas to provide reference for the tract-tracing images. Relevant structures to this study as well as reference landmarks are provided. Injections of biocytin to the AOE vesicles revealed labeling in the olfactory system and the telencephalon. Neurons with wide, thick cell bodies with a dendritic knob and cilia extending into the lumen of the accessory olfactory organ were evident (Figure 2A). Labeled cells in the MOE showed a retrograde connection from the AOE, however this could be due to leakage of dye from the AOE to olfactory nerve fibers rather than a physical connection between the AOE and MOE. Tall, thin neurons were labeled in the basal lamellae of the MOE (Figure 2B). Labeled cells lining the MOE were pseudo-stratified ciliated columnar cells and those lining the AOE were ciliated cuboidal cells. The dorsal half of the olfactory nerve was more strongly labeled than the ventral part (Figure 2C). Anterogradely, labeled fibers and cells were observed in the medial olfactory bulb, at the ventral aspect of the DTN as well as the preoptic and striatum area (Figure 2D, E, F). Coarse, short fibers were visible in the DTN and cell bodies were seen at the ventral DTN (Figure 2G). A bundle of thick fibers was seen between the medial pallium and the DTN, as well as cell bodies in the dorsal pallium and ventral DTN (Figure 2H). The dorsal pallium (Figure 2I) showed a grouping of coarse fibers and some cell bodies. In summary, the AOE has connections to the MOE, the olfactory bulb, the DTN and pallia, and indirectly to the rostral hypothalamus.

### DTN connects to the AOE and the hypothalamus

**Figure 3:**
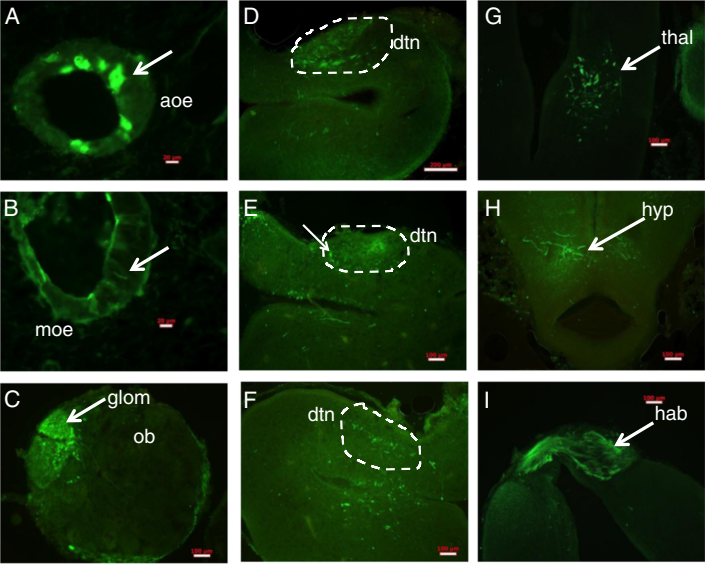
**Anterograde and retrograde connections of the lamprey dorsomedial telencephalic neuropil (DTN).** Biocytin injections were made to the DTN and representative pictures are shown here. **A**, following dye loading, round or ovoid shaped cells are labeled in AOE vesicles (red arrow). Scale bar: 20 μ **B**, olfactory sensory neurons with long thin cell bodies are seen in the main olfactory epithelium (red arrow). Scale bar: 20 μm. **C**, medial glomerular territories are labeled. Scale bar: 100 μm. **D**, the DTN has a dense population of fibers and a smaller population of coarse thick fibers at the ventral portion, as well as some thick short fibers in the striatum. Scale bar: 200 μm. **E**, the caudal aspect of the DTN has a sparse population of short fibers, both coarse and thin. Labeled cells are dorsoventrally oriented, located proximate to the DTN (arrow) **(F)** and visible throughout the entire rostrocaudal extent of the DTN. **G**, coarse, thick fibers are labeled in the thalamus. Scale bar = 100 um. **H**, coarse, thick fibers are seen bilaterally in the hypothalamus. Scale bar = 100 um. **I**, the habenula is densely labeled with thin and thick fibers. aoe: accessory olfactory epithelium; dtn: dorsomedial telencephalic neuropil; glom: glomerular territory; hab: habenula; hyp: hypothalamus; moe: main olfactory epithelium; ob: olfactory bulb; thal: thalamus.

Injections of biocytin to the DTN revealed a reciprocal labeling of cells in the AOE as well as direct projections to various regions of the telencephalon. Retrograde labeling revealed round cells in the AOE (Figure 3A) and tall elongate cells in the MOE (Figure 3B). Short fibers and cell bodies were labeled in the medial olfactory bulb (Figure 3C), similar to the results shown in Derjean et al., 2010 [28]. The rostral DTN was densely labeled with fibers (Figure 3D, E). Within the DTN were some cell bodies oriented dorsoventrally with at least 1 process extending dorsally toward the DTN (Figure 3E; see Additional file 1 for greater detail). At the caudal end of the DTN, the fiber population was smaller than at the rostral end. As well, the fibers were short, coarse and grouped at the dorsal part of the DTN (Figure 3F). The thalamus had short, coarse fibers bilaterally located (Figure 3G). The rostral hypothalamus had short coarse fibers and some cell bodies bilaterally at the level of the mammillary recess (Figure 3H). In the habenula, a mixed population of thin and thick fibers was seen (Figure 3I). In summary, sea lamprey DTN has connections to the AOE as well as multiple integrative centers in the telencephalon (dorsal pallium, lateral pallium, thalamus and hypothalamus).

### MOE and AOE have virtually identical gene expression profiles

**Figure 4:**
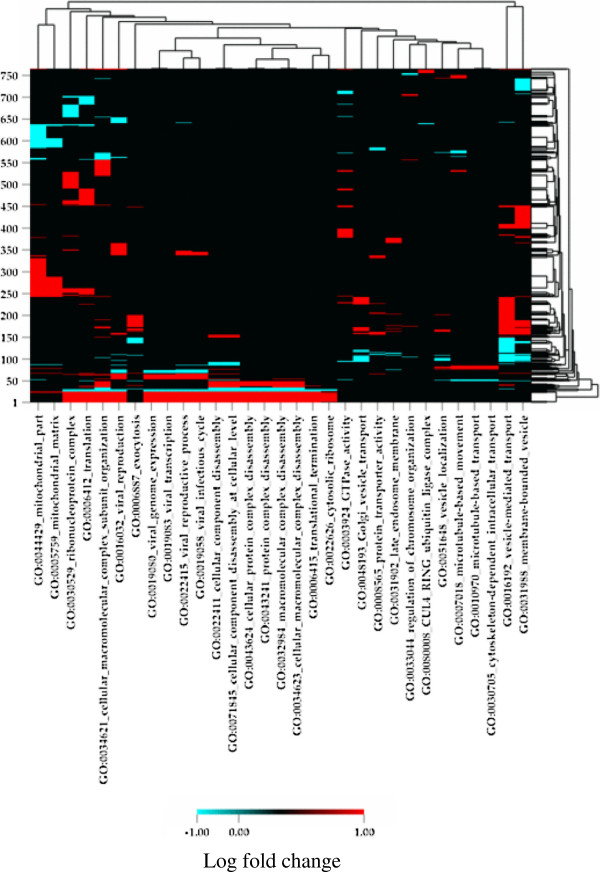
**Comparisons of the transcriptomes (accessory vs. main olfactory epithelium) using gene ontology (GO) analyses.** Transcriptomes were obtained using Illumina DGE sequencing technology. X-axis represents the GO categories and Y-axis represents gene clusters. Color scale represents the Log_2_ (transcript number in accessory/transcript number in main olfactory epithelium). X-axis: 1.GO0044429 mitochondrial part, 2.GO0005759 mitochondrial matrix, 3.GO0030529 ribonucleoprotein complex, 4.GO0006412 translation, 5.GO0034621 cellular macromolecular complex subunit organization, 6.GO0016032 viral reproduction, 7.GO0006887 exocytosis, 8.GO0019080 viral genome expression, 9.GO0019083 viral transcription, 10.GO0022415 viral reproductive process, 11.GO0019058 viral infectious cycle, 12.GO0022411 cellular component disassembly, 13.GO0071845 cellular component disassembly at cellular level, 14.GO0043624 cellular protein complex disassembly, 15.GO0043241 protein complex disassembly, 16.GO0032984 macromolecular complex disassembly, 17.GO0034623 cellular macromolecular complex disassembly, 18.GO0006415 translational termination, 19.GO0022626 cytosolic ribosome, 20.GO0003924 GTPase activity, 21.GO0048193 Golgi vesicle transport, 22.GO0008565 protein transporter activity, 23.GO0031902 late endosome membrane, 24.GO0033044 regulation of chromosome organization, 25.GO0080008 CUL4 RING ubiquitin ligase complex, 26.GO0051648 vesicle localization, 27.GO0007018 microtubule-based movement, 28.GO0010970 microtubule-based transport, 29.GO0030705 cytoskeleton-dependent intracellular transport, 30.GO0016192 vesicle-mediated transport, and 31. GO0031988 membrane-bounded vesicle.

The first attempt to discover gene categories that differed between the MOE and AOE by 2 fold (log_2_ 2 = 1.0) failed to show any differences in gene expression between the two epithelia. Therefore, the threshold for differential gene expression was lowered (log_2_ 1.414 = 0.5) which corresponds to a 1.414 fold change in expression. 31 of 11,225 gene ontology (GO) categories were shown to be differentially expressed between the two epithelia, which represent less than 0.3% of the GO categories compared. A heat map of the 31 GO categories changed is shown in Figure 4. The majority of GO category differences are due to cell maintenance or receptor trafficking (e.g. GO0010970: microtubule based transport or GO0048193: Golgi vesicle transport).

### Expression of chemoreceptor genes is sexually dimorphic

**Figure 5:**
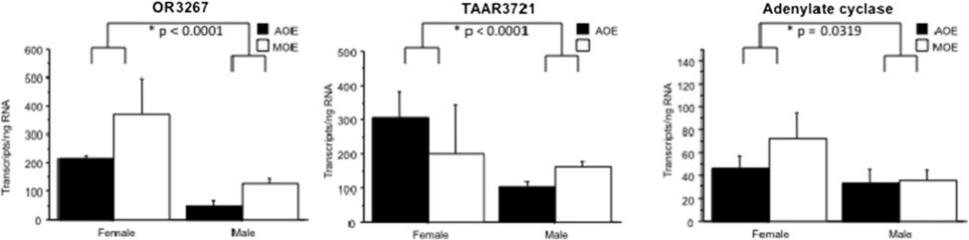
**OR 3267, TAAR 3721 and adenylate cyclase are expressed significantly higher in adult female than in adult male sea lampreys.** SYBR green real time quantitative PCR reveals olfactory receptor 3267 (p < 0.0001), trace amine-associated receptor 3721 (p < 0.0001) and adenylate cyclase (p = 0.0319) are expressed significantly higher in adult female lampreys than in adult males.

**Figure 6:**
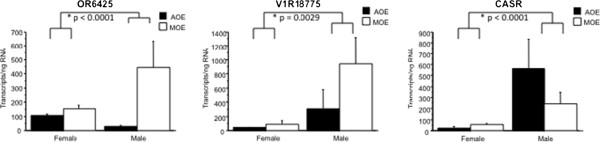
**OR 6425, V1R 18775 and CASR are expressed significantly higher in adult male lampreys than in adult female lampreys.** SYBR green real time quantitative PCR reveals olfactory receptor 6425 (p < 0.0001), vomeronasal type-1Receptor 18775 (p = 0.0029) and a calcium sensing receptor (p < 0.0001) are expressed significantly higher in adult male than in adult female sea lampreys.

Sequences generated from high-throughput sequencing were aligned to the mouse RefSeq mRNA database. Using these sequences in combination with those identified by Libants et al. [20], representative chemoreceptor and chemoreceptor-related genes were selected to confirm the Solexa sequencing results and to further examine the chemoreceptor gene expression of the MOE and AOE. Real time quantitative PCR confirmed expression of six chemoreceptor and chemoreceptor-related genes (OR 3267, OR 6425, TAAR 3721, V1R 18775, CASR and adenylate cyclase) in both the MOE and AOE. Collectively, MOE and AOE displayed a sexually dimorphic pattern in expression of CR and CR-related genes. OR 3267 (p < 0.0001), TAAR 3721 (p < 0.0001) and adenylate cyclase (p = 0.0319) were expressed higher in adult females than in males (Figure 5) while OR 6425 (p < 0.0001), V1R 18775 (p = 0.0029) and CASR (p < 0.0001) were expressed higher in adult males than in females (Figure 6). The expression levels of these genes did not differ between MOE and AOE.

## Discussion

This study discovered striking similarities between the tetrapod vomeronasal pathway and a lamprey accessory olfactory pathway containing the AOE and DTN, as shown in Table 1. GO analysis coupled with real-time quantitative PCR demonstrated that lamprey MOE and AOE gene expression profiles are similar. Lamprey AOE expresses all known families of lamprey chemoreceptor genes. Taken together, results suggest that the sea lamprey possesses a chemosensory accessory olfactory system.

**Table 1:** Comparison of main and accessory olfactory system components in rodent, frog, zebrafish and lamprey *note – teleost fish do not have a recognized vomeronasal organ nor an accessory olfactory bulb

		**MOE**			**MOE/AOE**	**AOE**		
		**Rodent**	**Frog**	**Lamprey**	**Zebrafish**	**Rodent**	**Frog**	**Lamprey**
Peripheral	Cell Type	Pseudostratified Ciliated Columnar [29]	Ciliated [30,31]	Pseudostratified Ciliated Columnar [32,33]	Pseudostratified Ciliated Columnar and Microvillous [34]	Pseudostratified Ciliated Columnar [35]	Microvillous [31]	Simple Ciliated Cuboidal [23,24,36]
Peripheral	Genes	OR, TAAR [10]	OR [37], V1R [9]	OR, TAAR, V1R [22]	OR [38], TAAR [39], V1R, V2R [17]	OR, V1R, V2R [10]	V2R [40]	OR, TAAR, V1R [15,22]
Peripheral	Projections	To MOB [41]	To MOB and AOB [31,42]	To MOB and DTN	To MOB [34]	To AOB [43]	To MOB and AOB [31,44]	To MOB and DTN
Central	Cell Type	Mitral cells [45]	Mitral cells [42,46]	Mitral cells [24,33]	Mitral cells [47,48]	Mitral cells [49]	Mitral cells [44,50]	Mitral cells
Central	Glomeruli	Yes [51]	Yes, [42,50]	Yes, loosely defined [52]	Yes [34,47,53,54]	Yes [43]	Yes [44]	Yes, loosely defined [52]
Central	Secondary Projections	To olfactory cortex [55]	To olfactory amygdala [56]	To pallial areas and hypothalamus [26,27]	To habenula/limbic system output pathway [54]	To amygdala (limbic area) and hypothalamus [57]	To piriform cortex [58]	To pallial areas and hypothalamus

Our neural tract-tracing results show direct projections from the AOE to the DTN. Injections of biocytin to the AOE revealed connections to the medial olfactory bulb similar to the results of Derjean et al., 2010 [28], the pallial areas of the telencephalon and the DTN. Labeling of cells in the MOE after injection to the AOE was unexpected as the MOE and AOE are anatomically separate, however, this may be due to piercing of olfactory nerve fascicles during injection, which are in close proximity to the AOE vesicles [23,24]. Alternatively, some AOE vesicles have been observed to be connected to the MOE by ducts at the ventrolateral aspect of the nasal capsule, though this assertion could be an artifact of the plane of sectioning [59]. Moreover, dye could have been transported anterogradely to the MOE from the AOE via the olfactory nerve axons that are in close proximity to the AOE. Injections of biocytin to the DTN revealed reciprocal connections with the AOE and the MOE. While the primary projections of the AOE to the DTN in lamprey are very similar to the primary projections of the VNO to the AOB in tetrapods, a difference is that in lamprey, the AOE has direct projections to the MOB [28]. In tetrapods, the MOE and VNO have segregated outputs to the MOB and AOB, respectively [57]. Therefore, the lamprey pathway is less segregated than those in adult tetrapods.

Interestingly, the lamprey system shares similarities with the system in developing tetrapods. Previous studies have already demonstrated anatomical evidence that MOE and AOE both project to the medial olfactory bulb and functional evidence that the medial olfactory bulb activates locomotor brain regions [28]. Our work builds on these findings via anterograde and retrograde tracings from the AOE and the DTN of lamprey to show partial segregation at the peripheral level. The vomeronasal system recently discovered in lungfish is also a less segregated system [14], as molecular markers for a VNO are expressed in the MOE. The question of the ancestral vertebrate condition with respect to olfactory projections (mixed or segregated outputs) requires further investigations.

Another similarity seen between the lamprey and tetrapod pathways is in their projections to higher centers. The lamprey DTN has direct projections to a putative amygdala homolog as well as the hypothalamus and thalamus. Dye injections to the DTN revealed labeling in the dorsal pallium, the hypothalamus and the thalamus. This confirms previous discoveries by Northcutt and Puzdrowski [26] who demonstrated DTN connectivity to the hypothalamus. Polenova and Vesselkin [27] also demonstrated connectivity of the DTN to the pallial areas of the telencephalon. Our work provides further information on the telencephalic pathways with respect to the main and accessory olfactory epithelia. The bi-directional connectivity between the medial pallium and striatum has been demonstrated in silver lamprey by Northcutt and Wicht [60]. Furthermore, the pallial areas are likely homologs of the tetrapod amygdala because of GABA-ergic projections from the medial pallium to the striatum [61]. Consequently, the pattern of projection of AOE to DTN to pallial areas and hypothalamus likely parallels the tetrapod vomeronasal pathway.

The pathway seen in our study flows from the AOE to the DTN to the pallial areas and the hypothalamus. In tetrapods, the MOE and VNO have anatomically distinct primary projections. The MOE projects primarily to the main olfactory bulb and the VNO projects to the accessory olfactory bulb. In mice, there is a further segregation of output from the VNO. Specifically, sensory neurons in the anterior and posterior VNO express V1R and V2R receptors, respectively, and project to the anterior and posterior AOB, repeating the anatomical division seen at the periphery [1,43]. Output neurons from the AOB in turn project to limbic areas of the brain including the amygdala and also to the hypothalamus and thalamus [1]. From the AOB, there are two distinct populations of output neurons that project to the rostral and caudal regions of the amygdala, which in turn project to rostral and caudal regions of the hypothalamus which mirrors the segregated inputs from the vomeronasal organ [57,62]. In sea lamprey, there is a convergence of output from the MOE and the AOE. Both the MOE and AOE have connections to the OB and the DTN, and so there is not a clear division of output from the MOE and AOE to their respective olfactory integration centers.

The sea lamprey AOE has cellular and molecular characteristics of an olfactory sensory epithelium. Since its discovery in *Petromyzon* by Scott in 1887 [23], AOE has been suggested to function as Jacobsen’s organ [23], nasal sac rudiments [63], part of the pituitary [64] and Bowman’s glands [65]. Recently, Ren et al. [24] demonstrated retrograde connectivity from the medial olfactory bulb to the AOE and concluded that the AOE and its projections are a distinct division within the olfactory pathway. Our data complements this conclusion by demonstrating anterograde connectivity from the AOE to the medial OB. In addition, we have shown reciprocal connectivity between the AOE and the DTN. Morphologically, the retrogradely labeled sensory neurons from both MOE and AOE in lamprey are ciliated. Molecular level analysis revealed further evidence that the lamprey AOE is a sensory epithelium. As expected, the overall gene categories expressed in MOE and AOE are virtually identical, furthering the case of the AOE as a chemosensory structure. Expression of chemoreceptor genes from all three of the families of chemoreceptor genes (ORs, TAARs and V1Rs) identified in the lamprey genome was confirmed [22]. In tetrapods, the VNO expresses V1Rs, V2Rs and ORs [4,8,10,66,67] while the MOE expresses ORs, TAARs and V1Rs [9]. While the MOE and VNO are anatomically separate in tetrapods, there is overlap with respect to chemoreceptor gene expression, secondary projection pathways and neural connectivity [8,11,40,68]. The similarities in chemoreceptor gene families expressed in lamprey MOE and AOE may be explained by the status of the lamprey as a basal vertebrate [69,70]. Moreover, during embryological development, the MOE and AOE of vertebrates both arise from the olfactory placode [71,72]. At the neural circuit level, as well as the molecular level, it appears that the lamprey dual system is not as segregated as the tetrapod dual olfactory system.

Chemoreceptor genes were found to have a sexually dimorphic pattern of expression in lamprey MOE and AOE. In vertebrates, sexually dimorphic gene expression is usually linked to sex determination. For example, in rainbow trout, sox9a1 is expressed in male gonads and cyp19a1 is expressed in female gonads [73]. In the sea lamprey, the gene expression pattern observed in this study may be related to its sexually dimorphic behavior. While both males and females can detect the pheromone 3-keto petromyzonol sulfate (3 kPZS), only females show a strong locomotor response [74]. However, this speculation requires further examinations.

## Conclusion

Anatomical and molecular evidence shows that the sea lamprey has a primordial accessory olfactory system that may serve a chemosensory function.

## Methods

### Experimental animal

Migrating adults (n = 93) were obtained from the St. Mary’s River in Sault Ste. Marie, Michigan from the Hammond Bay Biological Station with mean length ± s.d. (48.3 cm ± 0.4 cm) and mean weight ± s.d. (237.4 g ± 5.0 g). Animals were handled according to guidelines provided by the Institutional Animal Care and Use Committee at Michigan State University.

### Neural tract tracing

Animals were euthanized in tricaine methanesulfonate (MS-222, 100 mg/L, Sigma). The olfactory epithelium and brain were rapidly exposed by dorsal dissection, removing any surrounding muscle or cartilage. The tissue was rinsed in aerated cold Ringer’s solution (pH 7.4) with the following composition: 130 mM NaCl, 2.1 mM KCl, 2.6 mM CaCl_2_, 1.8 mM MgCl_2_, 4 mM HEPES, 4 mM dextrose and 1 mM NaHCO_3_. Glass capillaries with a diameter of 50 μm were filled with 2 μl of 2% biocytin [in 0.1M phosphate buffer saline (PBS), pH7.2] and inserted into either multiple accessory olfactory vesicles or the DTN (see Additional file 2), and the tracer was applied to the lesion. Tissue was rinsed and incubated in lamprey Ringer’s for 10 minutes before being placed in a flow-through chamber held at 7°C. The tissue was continuously perfused with cold aerated Ringer’s solution during the entire incubation period. After 4 hours, the tissue was fixed in 4% paraformaldehyde in 0.1 M PBS (pH 7.4). Tissue was then immersed in Sakura Tissue-Tek O.C.T. compound (VWR) and frozen with a combination of liquid nitrogen and dry ice. Thin sections (20 μm) were collected on Superfrost Plus slides (VWR) and stored at −20°C. Slides were washed in 0.1 M PBS (pH 7.4) and biocytin signal was visualized by addition of Alexa 488 Streptavidin (1:100, Invitrogen). Slides were examined on an upright Zeiss Axioskop 2, equipped with fluorescence and a CCD camera. Images were captured using Axiovision software (Zeiss). Samples with clear leakage from the intended injection site were rejected.

### Laser Capture Microdissection (LCM) and mRNA-Seq preparation

Olfactory organs from mature males and females were dissected out, embedded in O.C.T. compound and frozen with a combination of dry ice and liquid nitrogen. Seven-μm frontal sections were collected on non-charged glass slides (VWR) and stored at −80°C. Slides were then passed through an ascending alcohol series and rinsed with xylene to dehydrate the tissue and remove the alcohol. Slides were then viewed under an inverted Nikon Eclipse microscope outfitted with the Arcturus Pixcell II/e Laser Capture Microdissection System and Arcview software (Arcturus). The MOE and the AOE are not distinguishable with the naked eye, but are easily distinguished when viewed under a microscope (data not shown). Cells from the MOE and AOE were lifted under the following conditions (duration: 20.0 ms, repeat: 0.4 s, spot size: 7.5 μm, power: 100 mW). Because of the anatomical separation of the MOE and AOE, we were absolutely sure that we were lifting cells from the appropriate epithelium. RNA was extracted using TRIZOL reagent (Invitrogen) and stored at −80°C. Quality of samples was verified using an Agilent 2100 Bioanalyzer before submission for high-throughput sequencing.

### GO analyses

MOE and AOE RNA samples were sequenced at the Michigan State University Research Technology Support Facility, using the Illumina DGE kit according to manufacturer’s instructions. 64,141,260 reads were obtained and 58% (37,785,187) passed a quality filter. The filtered reads were aligned, using Bowtie software [75], to our assembly of the sea lamprey transcriptome to obtain transcript expression count information for each lane, which were then quantile-normalized. The transcriptome assembly was, in turn, aligned to mouse RefSeq protein sequences, providing a putative orthology with which mouse protein annotations were assigned to corresponding lamprey transcripts, and these annotations were combined with transcript expression counts to infer expression information for putative lamprey-mouse orthologs. This information was used to infer putative ortholog differential expression between MOE and AOE. Using inferred expression ratios, significantly enriched or depleted gene ontology categories were identified, with the help of GoMiner software [76].

### SYBR green real-time quantitative PCR

Cells from MOE and AOE of six individuals (four male, two female) were collected using LCM. RNA from these cells were extracted and used for real-time quantitative PCR (methods followed Chung-Davidson et al. [77]). Solexa DGE reads were aligned to the mouse refseq mRNA database [78] and chemosensory and chemosensory-related genes were selected from the putative mouse orthologs. Only full-length, intact sequences were used for primer design using Primer Express software (Applied Biosystems) (Table 2). The sea lamprey genome does not possess vomeronasal type-2 receptors (V2R), but does contain calcium-sensing receptors (CASRs), which are V2R-like (Libants et al., 2009). The genes monitored were: OR 3267, OR 6425, TAAR 3721, V1R 18775, CASR and adenylate cyclase.

**Table 2:** Primers used for SYBR green qPCR

**GENE**	**FWD (SENSE)**	**REV (SENSE)**	**REV (5’-3’)**
OR3267	aaccgggctgagcaagaac	cgagggagcgagaaacttca	tgaagtttctcgctccctcg
OR6425	gaagaacatctgtgccatgca	gcagaacgtcgcgtcctt	aaggacgcgacgttctgc
TAAR3721	tctgcagctgcctgaagtagag	ccatcgcgggcaaca	tgttgcccgcgatgg
V1R18775	attggcacgtgtcacatgaga	gagagaacgcgaggcttatcag	ctgataagcctcgcgttctctc
CASR	ttttgaccaagatgcaagacaag	cccgccagcccttttt	aaaaagggctggcggg
AC9	cgccataggtatccacatcttca	tggcccaccttgaggaaag	ctttcctcaaggtgggcca
GP	ccaggccagggaaatgc	tgagctgaggcaagaagtaatcag	ctgattacttcttgcctcagctca

## Competing interests

The authors declare that they have no competing interests.

## Authors’ contributions

SC performed neural tract-tracing, LCM and qPCR. YWCD assisted with qPCR. SVL assisted with qPCR and sequence alignments. KGN performed GO categorization and sequence alignments. MK assisted with LCM. CTB assisted with GO categorization and sequence alignments. WL and YWCD conceived this study. SC, YWCD and WL drafted the manuscript. All authors read and approved the final manuscript.

## Supplementary Material

Additional file 1: Figure S1 DTN has cells and fibers. Description of dataset - A, Coarse fibers are seen at the ventral border of the DTN. Scale bar = 100 μm. B, A single cell in the DTN is dorso-ventrally oriented with dendrites extending dorsally. Scale bar = 20 μm. dtn: dorsomedial telencephalic neuropil. Click here for file

Additional file 2: Figure S2 Biocytin injection to dorsomedial telencephalic neuropil. Description of dataset - A, Sea lamprey brain exposed in the cranium. Injection site dorsal and medial at the margin of the olfactory bulb and telencephalon (blue dot arrow). B, Lesion in the dorsomedial telencephalic neuropil. Click here for file
