# Supplementary figures and images for: The sea lamprey has a primordial accessory olfactory system
Source: BMC Evol Biol. 2013 Aug 17;13:172. doi: 10.1186/1471-2148-13-172 (PMC3765145; doi:10.1186/1471-2148-13-172)

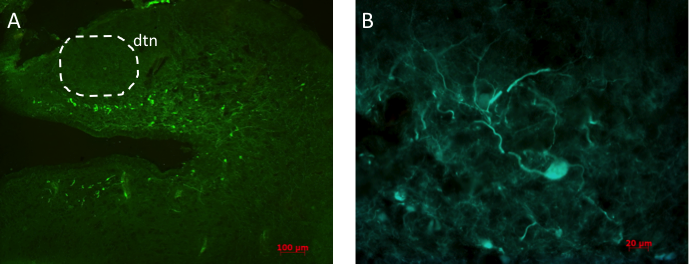

Supplement: Additional file 1: Figure S1 — DTN has cells and fibers. Description of dataset - A, Coarse fibers are seen at the ventral border of the DTN. Scale bar = 100 μm. B, A single cell in the DTN is dorso-ventrally oriented with dendrites extending dorsally. Scale bar = 20 μm. dtn: dorsomedial telencephalic neuropil. [file 1471-2148-13-172-S1.docx]
