# Supplementary material for: The sea lamprey has a primordial accessory olfactory system
Source: BMC Evol Biol. 2013 Aug 17;13:172. doi: 10.1186/1471-2148-13-172 (PMC3765145; doi:10.1186/1471-2148-13-172)
Supplement: Additional file 2: Figure S2 — Biocytin injection to dorsomedial telencephalic neuropil. Description of dataset - A, Sea lamprey brain exposed in the cranium. Injection site dorsal and medial at the margin of the olfactory bulb and telencephalon (blue dot arrow). B, Lesion in the dorsomedial telencephalic neuropil. [file 1471-2148-13-172-S2.docx]

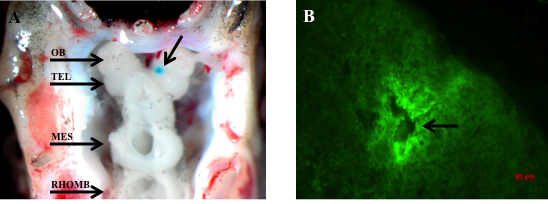


**Figure S2:** Biocytin injections to dorsomedial telencephalic neuropil and accessory olfactory epithelium. **A**, Sea lamprey brain exposed in cranium. Injection site at dorsal and medial at the margin of the olfactory bulb and telencephalon (blue dot/red arrow). **B**, Lesion in lateral DTN shown. Fluorescence is shown around the lesion, indicating it as the site of injection of biocytin.
